# Activation of LacZ gene in *Escherichia coli* DH5α via α-complementation mechanism for β-galactosidase production and its biochemical characterizations

**DOI:** 10.1186/s43141-020-00096-w

**Published:** 2020-12-02

**Authors:** Ahmed A. Hamed, Mohamed Khedr, Mohamed Abdelraof

**Affiliations:** 1 Microbial Chemistry Department, Genetic engineering and Biotechnology research Division, National Research Centre, El-Buhouth St, Dokki, Cairo 12622 Egypt; 2 Department of Botany and Microbiology, Faculty of Science, Al-Azhar University, Nasr City, Cairo Egypt

**Keywords:** β-Galactosidase, *Escherichia coli* DH5α, Trans-conjugation, α-Complementation, Purification

## Abstract

**Background:**

Plasmid propagation in recombination strains such as *Escherichia coli* DH5α is regarded as a beneficial instrument for stable amplification of the DNA materials. Here, we show trans-conjugation of pGEM-T cloning vector (modified Promega PCR product cloning vector with *tra* genes, transposable element (Tn5)) and M13 sequence via α-complementation mechanism in order to activate β-d-galactosidase gene in DH5α strain (non-lactose-fermenting host).

**Results:**

Trans-conjugation with pGEM-T allows correction of LacZ gene deletion through Tn5, and successful trans-conjugants in DH5α host cells can be able to produce active enzyme, thus described as lactose fermenting strain. The intracellular β-galactosidase was subjected to precipitation by ammonium sulfate and subsequently gel filtration, and the purified enzyme showed a molecular weight of approximately 72-kDa sodium dodecyl sulfate-polyacrylamid gel electrophoresis. The purified enzyme activity showed an optimal pH and temperature of 7.5 and 40 °C, respectively; it had a high stability within pH 6–8.5 and moderate thermal stability up to 50 °C.

**Conclusion:**

Trans-conjugant of *E. coli* DH5α- lacZ∆M15 was successfully implemented. UV mutagenesis of the potent trans-conjugant isolate provides an improvement of the enzyme productivity. The enzymatic competitive inhibition by d-galactose and hydrolysis of lactose at ambient temperatures could make this enzyme a promising candidate for use in the dairy industry.

## Background

β-Galactosidase or lactase (EC 3.2.1.23) is the enzyme that catalyzes the conversion of lactose to monosaccharide sugars. People with lactose intolerance are unable to make enough lactase enzymes which in turn causes the inability to consume dairy products [1–4].

The β-galactosidase enzyme has a broad utilization in food-processing industries such as hydrolysis of lactose in dairy or its derived products [5]. This enzyme also has many applications in a dairy product which plays an important role to avoid lactose crystallization, enhance flavor, boost the solubility of the milk product, and produce galacto-oligosaccharides for use in probiotic foods [6–8]. Furthermore, the production of colored products by this enzyme during chemical reaction has gained a great attention from researchers in the molecular biology field [5, 9].

Many sources such as plants, animal cells, and microorganisms have been investigated for their ability to produce valuable metabolites [10–12]. Recently, microorganisms have proved to be excellent source for production of several commercial enzymes with a wide variety of applications [13–15]. However, during the last several decades, bacteria are preferred as a source for several commercial enzymes such as β-galactosidase due to many advantages such as higher productivity and lower costs.

Indeed, until now, the kinetic properties of β-galactosidase used in the dairy industry having some limitations. One of them is the inhibition of β-galactosidase caused by the hydrolysis-formed product d-galactose which was regarded as a big barrier to its utilization in the industrial sector [2, 16]. Therefore, it is of great economic interest to explore a new source to generate β-galactosidase with improved processing characteristics for their utilization in dairy industries.

Recently, the use of recombinant DNA to convey and optimize the production and characteristics of bacterial enzyme has gained a great attention from researchers [2, 17, 18]. This strategy significantly extends the variety of prospective applications for β-galactosidase in the industry through increasing the enzyme’s manufacturing, optimizing the β-galactosidase enzyme’s productivity, and giving it new characteristics [19, 20]. One of these strategies is bacterial conjugation could be used to increase the bacterial β-galactosidase production. This process also known as horizontal gene transfer (HGT) is one the most widespread mechanisms for bacterial evolution [21]. The recombination of genes from a donor bacterium and genes of a recipient bacteria leads to the evolution of a new recombinant bacterium with a new genetic makeup and differs completely in characters from the two parent bacteria [22].

Although *Escherichia coli*’s β-galactosidase industrial use is restricted by the reality that it is not deemed safe for food applications, it is still useful and accessible for analytical purposes commercially [18].

*Escherichia coli* DH5α is a common laboratory bacterium engineered to maximize transformation efficiency; they are used extensively in recombinant DNA technology such as cloning and synthetic biology applications [23]. One feature of *E. coli* DH5α is the presence of three mutations, one of these mutations is the lacZM15 mutation, which deactivates LacZ activity in the *E. coli* DH5α producing an inactive form of β-galactosidase [24]. However, the activation of β-galactosidase enzyme of the *E. coli* DH5α can be achieved through α-complementation mechanism by introducing a plasmid carrying a LacZ alpha subunit into the *E. coli* DH5α strain, which therefore complements the truncated LacZ gene and produces an active β-galactosidase enzyme [23]. Here, we study the α-complementation mechanism for activation of a LacZ gene in the *E. coli* DH5α (recipient cells) using *E. coli* LK111 with (pGEM-T Vector) (donor cells) recombined with Complete LacZ; this plasmid had a high copy number vector and contains T7 and SP6 RNA polymerase promoters flanking a modified multiple cloning region that shifts to be not in the α-peptide coding region of the enzyme β-galactosidase. pGEM-T vector contains multiple restriction sites within the multiple cloning region. These restriction sites allow for the release of the insert by digestion with a single restriction enzyme (Promega, pGEM®-T, and pGEM®-T Easy Vector Systems). Furthermore, UV mutagenesis was carried out to improve the enzyme productivity. The biochemical characterizations of the purified enzyme and its potential application in lactose bioconversion process were also evaluated.

## Methods

### Strains, plasmids, and media

#### Strains

*E. coli* DH5α (dlacZ Delta M15 Delta(lacZYA-argF) U169 recA1 endA1 hsdR17(rK-mK+) supE44 thi-1 gyrA96 relA1

*E. coli* LK111 (F' lac-pro Δ(lacZ)M15/thi-1 thr-1 leuB6 tonAlacI- Δ(lacZ)M15 lacY+ supE44 P1s) with (pGEM-T) [25, 26] was modified in this study by inserting kanamycin and rifampicin resistance genes instead of ampicillin resistance gene as a selectable marker; also, this plasmid was genetically engineered by adding *tra* genes, M13 sequence. These two bacterial strains were kindly obtained from Applied Microbial Genetics Lab, Cytology and Genetics Dept., National Research Centre (NRC), Dokki, Egypt.

#### Media

1. *Laura Bertani Broth* (LB broth) is specific for the growth and maintenance of *E. coli* strains in molecular microbiology. It is composed of 10 g Bacto-tryptone, 5 g yeast extract, and 5 g NaCl dissolved in 1000 mL of dH_2_O (distilled water) and pH 7.02.
2. *Macconkey Agar medium* is a ready medium, used to differentiate between lactose fermenting *E. coli* strains. This medium is efficient in detecting strains with (LacZ) which encodes β-galactosidase enzyme. It is composed of 17 g peptone (pancreatic digest of gelatin), 3 g proteose peptone (meat and casein), 10 g lactose monohydrate, 1.5 g bile salts, 5 g sodium chloride, 0.03 g neutral red, 0.001 g crystal violet, and 13.5 g agar for 1 L.
3. *Minimal medium* (M9) is used for detecting recombinant strains which can be grown on lactose as only carbon source due to its β-galactosidase activity and composed of 12.8 g Na_2_HPO_4_, 3 g KH_2_PO_4_, 0.5 g NaCl, 10 g NH_4_Cl, 0.49 g MgSO_4_.7H_2_O, 0.015 g CaCl_2_.2H_2_O, 0.01 g FeSO_4_.7H_2_o, 0.01 g thiamine, and 2 g lactose per liter, and 20.0 g agar.

#### Bacterial trans-conjugation

The overnight cultures of recipient and donor strains were diluted 50-fold in LB medium. Both recipient and donor strains were incubated at 37 °C under shaking condition until reaching O.D. 0.40–0.60 at 600 nm. Recipient and donor cultures were mixed in a ratio of 1:10 (v/v). Trans-conjugants were selected on medium supplemented with rifampicin (Rif) and kanamycin (Kn) in 5 mg/ml from both

### UV mutagenesis

To induce the mutations in trans-conjugant *E. coli* isolate, ultraviolet (UV) irradiation was carried out according to the modified method of [27], where the bacterial cell suspension was prepared from overnight cultures by shaking for 5 min. Bacterial cells were exposed to UV with 254 nm using Philips T-UV-30 W lamp type number 57413 p/40 at a distance of 20 cm for different time interval (2, 5, 10, 15, 20, and 25 min). After irradiation, the treated cultures were protected from light by keeping in a dark place for 1 h. One milliliter of suitable dilution from treated cells was plated on minimal M9 with 10 g lactose and LB supplemented with 10 g lactose [28].

### Genomic DNA extraction

Alkaline Method Kit separated genomic DNA, and plasmid was modified as described by [29]. In an Eppendorff tube, 1.5 ml was taken from an overnight culture, centrifuged for 1 min at 8000×*g* to retain pellets. Three solutions were used in this method; the first one responsible for lysing the cellular membranes and cell wall called lysis solution A, 250 μl of solution A added (lysozyme solution mg/ml lysozyme, 50 mM glucose ,10 mM EDTA, 25 mM Tris-HCl, pH 8.0. Then, by shifting up and down three times, the second solution aids in membrane breakdown, about 250 μl solution B was added (SDS solution 0.2 N NaOH, 1% sodium dodecyl sulfate (SDS)) then blended. The solution C was the last high salt solution sodium acetate (pH 4.8) which was then added to 250 μl and centrifuged at 13,000×*g* for 5 min [30].

### Partial amplification of β-galactosidase gene

Beta-gal gene coding functional Beta-d-galactosidase enzyme was detected and amplified by two specific primers F-primer (Gal-F) 5-TTCCATGTTGCCACTCGC-3 and R-primer (Gal-R) 5-ATGATGCTCGTGACGGTTAA-3. The PCR mixture was as follows: Dream Taq buffer 2.5 μl, DNA template 6 μl (40 ng), Taq DNA polymerase 2.5 μl (2.5 U), dNTPs 1 μl (0.2 mM), MgCl2 2.5 μl (2.5 mM), primers (each one 1 μl) with concentration 20 pmol, and deionized H2O 8.5 μl.

### Enzyme assay and protein estimation

Enzyme activity was assayed by measuring the amount of *o*NP (*O*-nitrophenolate) liberated. Briefly, the reaction was initiated by adding 25 μl enzyme to 225 μl of *ortho*-nitrophenyl-β-galactoside (*o*NPG, 5 mM) in 50 mMMcIlvaine buffer (pH 6.5) which was incubated at 40 °C for 10 min. The reaction was stopped by adding 750 μl of 2 M Na_2_CO_3_ solution, and the absorbance of the mixture at 410 nm was then measured. One unit of enzyme activity was referred to the amount of enzyme releasing 1 μmol of *o*NPG per minute under the defined assay conditions. In order to determine the β-galactosidase activity toward lactose as a natural substrate, the reaction assay was performed as described by [2] with minor modification. Briefly, 500 μl of a lactose stock solution (100 mM) was added to 480 μL of 50 mM of McIlvaine buffer (pH 6.5) and incubated at 40 °C for 5 min. After that, 200 μl of enzyme solution was added to their action mixture for 15 min. The reaction was terminated by boiling at 99 °C for 5 min to inactivate the enzyme. After being cooled, d-glucose liberated in the reaction mixture was quantified using the Glucose Assay Kit [31]. One unit of enzyme activity was defined as amount of β-galactosidase liberating 1 μmol of d-glucose per minute under the defined assay condition.

Protein concentrations were determined by the Bradford method [32] using bovine serum albumin (BSA) as the standard.

### β-Galactosidase purification

The culture broth of *E. coli* DH5α was centrifuged at 10000×*g* for 15 min at 4 °C, and the resulting cell pellet was subsequently resuspended in McIlvaine buffer (50 mM, pH 6.5). The cell suspension was subjected to disruption by sonication in ice using a Sonicator (Vibra-Cell 72405) for 15 min with a 10 s on/10 s off pulse cycle at 60 W, and the cell debris were discarded by centrifugation at 10,000×*g* for 15 min at 4 °C to obtain the crude cell-free extract. The obtained extract solution was saturated with 60–80% ammonium sulfate and kept at 4 °C to precipitate the protein. The precipitate was collected after centrifugation and then dissolved in 50 mM McIlvaine buffer (pH 6.5) and dialyzed for 24 h against the same buffer with three times at equal time intervals. Dialyzed enzyme preparation was applied in a Sephadex G-100 column equilibrated with 50 mMMcIlvaine buffer (pH 6.5); the enzyme was eluted with the same buffer at a flow rate of 0.5 ml · min^−1^. Each fraction of 5 ml was collected and assayed for β-galactosidase. Total protein content was determined before and after dialysis. Protein concentrations were measured by A_280_ and A_260_ nm using the method described by [33]. The active fractions containing β-galactosidase activity were pooled, concentrated, and checked by sodium dodecyl sulfate-polyacrylamid gel electrophoresis (SDS-PAGE) [34]

### Effect of temperature, pH, and salt on β-galactosidase activity

The optimal temperature of the enzyme was performed by determination of the activity at different temperatures ranging from 30 to 80 °C. Study of the effect of pH on the activity was performed under various pH buffers, McIlvaine (50 mM, pH 4–6), phosphate (50 mM, pH 7–8), and carbonate (50 mM, pH 9–10). The effect of ionic strength on β-galactosidase activity was assessed by incorporation different concentrations of NaCl (0–4.0 M) with the enzyme reaction mixture, using 5 mM *o*NPG as substrate at 40 °C.

### Effect of temperature and pH on β-galactosidase stability

To measure the β-galactosidase stability against temperature and pH, the enzyme was incubating at different temperatures (30–80 °C for 2 h) and the residual activity was determined every 20 min. The enzyme was also incubated with different pH buffers comprised between 4.0 and 10.0 for 14 h at 4 °C prior to enzymatic activity assayed. Then, the relative β-Galactosidase activities were assayed under the optimal conditions described above.

### Effect of saccharides, metal ions, and other chemical compounds on β-galactosidase activity

The effects of d-glucose and d-galactose on the enzyme activity were estimated by determining the enzymatic activity in the presence of various concentration of these saccharides (5–250 mM d-glucose or d-galactose) at 40 °C for 10 min in 50 mM phosphate buffer (pH 7.5) using 5 mM *o*NPG as the substrate [35]. Determination of the inhibition type (competitive or non-competitive) by these saccharides was conducted based on non-linear Lineweaver and Burk reciprocal plot. On the other hand, the effect of metal ions (with chloride salt) and other chemical reagents on the enzyme activity was also carried out by incorporation of individual various cations (at 10 mM) or chemicals (at 5 mM) in the substrate-enzyme reaction. After enzymatic activity assayed, the residual activities were determined as described above, the enzymatic activity assayed without metal ions or chemicals was considered as a control 100%.

### Effect of organic solvents on β-galactosidase activity and stability

Additionally, the effect of organic solvents (methanol, ethanol, and isopropanol) on the enzyme activity at concentrations of 5%, 10%, and 20% was determined. In addition, β-galactosidase was incubated with each of organic solvents (at 10% and 20%, v/v) at 30 °C for 2 h under shaking at 180 rpm to measuring the enzymatic stability in organic solvent solutions [36].

### Substrate specificity and kinetic parameters of β-galactosidase activity

The substrate specificity of β-galactosidase was determined by measuring the enzyme activity toward different substrates (5 mM), involving oNPG, *p*NPG, d-lactose, xylose, and carboxymethyl cellulose (CMC). On the other hand, initial reaction rates were determined at various concentrations of both *o*NPG (0.5–25 mM) and lactose (20–360 mM) in 50 mM potassium phosphate buffer (pH 7.5) at 40 °C. The apparent maximum reaction velocity (*V*_max_) and the Michaelis constant (*K*_m_), turn over number (*K*_cat_), and *K*_cat_*/K*_m_ ratio were calculated using Lineweaver and Burk reciprocal plot.

### Lactose bioconversion by *E. coli* DH5α β-galactosidase

The appropriate amount of purified β-galactosidase was incubated with 1 ml of lactose solution (5%, w/v), at 40 °C in 50 mM potassium phosphate buffer (pH 7.5) with constant stirring (500 rpm). Samples were withdrawn at different times and heated in boiling water for 5 min, and the composition of sugar mixtures was then analyzed by high-performance liquid chromatography (HPLC). d-Lactose, d-galactose, d-glucose, and galacto-oligosaccharides (tri- and tetra-saccharides) were used as the authentic reference sugars determined by Agilent Technologies 1100 series liquid chromatography equipped with an auto sampler and a refractive index detector. The analytical column was SCR-101 N. The mobile phase was deionized water, and the flow rate was 0.7 ml/ minute. The temperature of the oven was optimized to 40 °C. Prior to injection, samples were diluted and filtered through a 0.22-μm Nylon membrane in order to remove proteins that may cause interference in the analysis [35].

### Data analysis

The data represented in this work were expressed as the average ± standard deviation (SD) for *n* = 3 and were analyzed using SPSS-16

## Results and discussion

### Bacterial trans-conjugation and screening for β-galactosidase activity

One bacterial strain *E. coli* LK111 harbors genetically engineered pGEM-T (Promega PCR product cloning vector) with *tra* genes, and transposable element (Tn5) was used to perform bacterial trans-conjugation with *E. coli* DH5α producing an inactive form of β-galactosidase enzyme due to LacZ mutation. After bacterial trans-conjugation process was conducted, all isolates were tested for their ability to produce LacZ activity according to α-complementation mechanism.

In this way, all trans-conjugant recombinant cells were tested for their ability to grow in lactose minimal media agar plates, and only cells that have a functional beta-galactosidase enzyme can be indicated as colony-forming unit (CFU). Trans-conjugant *E. coli* DH5α- lacZ∆M15 with genetically modified pGEM-T plasmid carrying M13 showed positive productivity of β-galactosidase enzyme among other strains (data not shown). To measure the enzyme activity, all preselected positive trans-conjugants were grown in liquid fermentation medium and assayed for β-galactosidase activity using *ortho*-nitrophenyl-β-galactoside (*o*NPG). As can be seen in Table 1, eighteen isolates were reasonably positive producers, and among these, about five trans-conjugant isolates coded as Tra5, Tra10, Tra210, Tra222, and Tra257 showed the maximum β-galactosidase activity after 24 h. There was a significant difference in the enzyme activity between these trans-conjugant isolates and other trans-conjugant. The most potent trans-conjugant isolate Tra210 was selected for further analysis.

**Table 1:** Screening of β-galactosidase activity

Trans-conjugants	Protein (mg/ml)	Enzyme activity (U/ml)	Specific activity (U/mg)
Tra5	3.43 ± 0.16	109.33 ± 2.05	31.9 ± 2.19
Tra10	3.36 ± 0.047	101.83 ± 0.97	30.2 ± 0.37
Tra127	5.3 ± 0.08	71.33 ± 1.47	13.4 ± 0.14
Tra200	2.76 ± .047	31.26 ± 0.89	10.92 ± 0.22
Tra202	3.53 ± 0.094	41.3 ± 0.90	11.69 ± 0.54
Tra210	1.8 ± 0.081	77 ± 1.41	41.96 ± 0.97
Tra212	5.03 ± 0.094	90.93 ± 1.03	18.03 ± 0.32
Tra213	3.6 ± 0.16	40.47 ± 0.42	11.02 ± 0.24
Tra216	4.26 ± 0.094	110.97 ± 0.56	25.96 ± 0.47
Tra222	3.03 ± 0.12	100.55 ± 0.25	33.2 ± 1.41
Tra230	5.73 ± 0.092	81.08 ± 0.72	14.1 ± 0.16
Tra233	2.2 ± 0.081	20.9 ± 0.08	9.46 ± 0.32
Tra234	2.83 ± 0.047	30.77 ± 0.67	10.8 ± 0.40
Tra250	4.13 ± .091	40.54 ± 0.51	9.81 ± 0.24
Tra252	5.63 ± 0.047	70.92 ± 0.52	12.57 ± 0.2
Tra257	3.46 ± 0.091	111.36 ± 0.90	32.1 ± 0.88
Tra258	2.2 ± 0.081	19.81 ± 0.39	8.98 ± 0.43
Tra271	4.33 ± 0.047	51.41 ± 0.83	11.8 ± 0.16

### UV mutagenesis

The best trans-conjugant *E. coli* Tra210 was subjected to UV irradiation for different time intervals (2, 5, 10, 15, 20, and 25 min) to improve β-galactosidase enzyme yield through random mutation. Six trans-mutant isolates exhibited improvement in the enzyme productivity coded as MKUV-Tran 2, MKUV-Tran 10, MKUV-Tran25, MKUV-Tran35, MKUV-Tran44, and MKUV-Tran52. MKUV-Tran44 was the best producer either on agar assay or on *o*NPG colorimetric assay with 21 mm and 251.7 U/ml respectively (Table 2). MKUV-Tran44 was the best producer as it has a pointed mutation in the regulatory region of LacZ gene which leads to the overexpression of this gene. UV mutagenesis provides an improvement of the enzyme productivity with 3.2-fold more than the wild trans-conjugant isolate.

**Table 2:** Screening of β-galactosidase productivity for the best trans-conjugant isolate (Tra210) after UV mutagenesis

Mutants	Clear zone (mm)	Enzyme activity (U/ml)
MKUV-Tra2	17	170.9 ± 1.2
MKUV-Tra10	19.5	190.22 ± 0.88
MKUV-Tra25	15	80.5 ± 1.5
MKUV-Tra35	13	70.4 ± 0.66
MKUV-Tra44	21	251.7 ± 1.22
MKUV-Tra52	18	190 ± 1.4

### Amplification of β-galactosidase gene

**Fig. 1:**
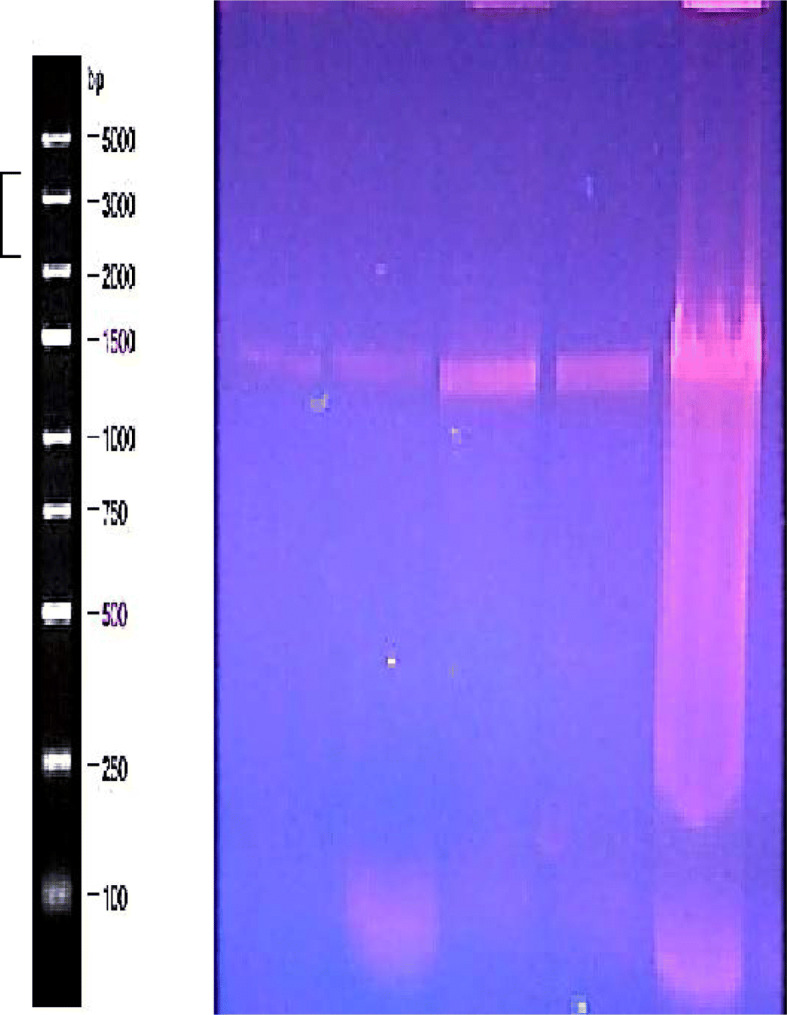
Agarose gel electrophoresis for PCR amplicon from LacZ gene of the best six producers bacterial isolates: from left to right MKUV-Tra2, MKUV-Tra10, MKUV-Tra25, MKUV-Tra35, MKUV-Tra44, and MKUV-Tra52 and all against 250 bp GENESTA DNA Ladder

The best producer trans-conjugated and mutant isolates (trans-mutants) were tested for PCR amplification of Beta-gal gene (LacZ) using two designed primers as described in the “Methods” section. PCR amplicons were extracted, purified from the gel (Fig. 1), and partially sequenced by a single FW primer as the gene size exceeded 3 kbps and needed more than a pair of primers for amplification and sequencing. The resulted sequences were analyzed against the most related organisms recorded on GenBank through DNA BLAST. Submission of these sequencing to GenBank under accession numbers MN172239, MN172240, MN172241, MN172242, MN172243, and MN172244 was carried out, and the molecular sizes of amplified beta-gal genes were 1590, 1590, 1590, 1576, 1576, and 1577 bps, respectively (Supplementary data Table 1). Consequently, DNA nucleotide sequence alignment of these trans-mutants was constructed online through clustal omega and edited through jalview software (Supplementary data Fig 1). Phylogenetic tree was also designed between LacZ gene sequences from all isolates (Supplementary data Fig 2). In addition, amino acids coded from DNA sequences were generated through MEGA 7 software (Supplementary data Fig. 3).

### Purification of β-galactosidase

**Fig. 2:**
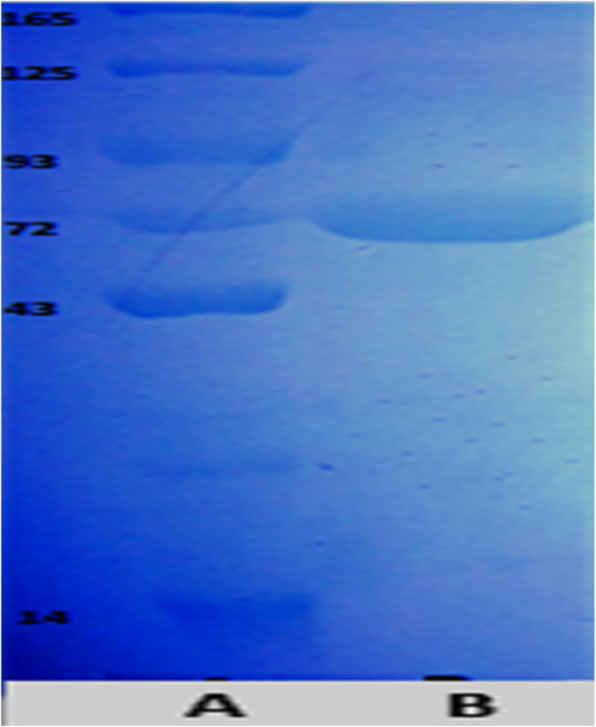
Molecular weight of *E. coli* MKUV-Tra44 β-galactosidase by electrophoretic analysis on 7.5% SDS-PAGE. **a** Molecular weight marker proteins. **b** Purified β-galactosidase

β-Galactosidase in the cell-free extract of the most potent trans-mutant strain (coded as M-KH-UV-Tra44) was purified to apparent homogeneity using a purification scheme based on a smaller number of purification steps. In many cases, the costs of purification operations can reach 80% of the total cost of production. Therefore, β-galactosidase purification using further column chromatography is undesirable for employments in the food industry and is attributed to the high costs of this process step [2, 37]. Thus, the enzyme was only purified using ammonium sulfate precipitation (60–80%), and the resultant protein was only applied in a Sephadex G-100 column (Table 3) for the functional property evaluation of the purified enzyme. In this way, after a fractionated ammonium sulfate precipitation of the cell-free extract, the purification fold was reached to 3.07-fold with 159.3 ± 0.81 units/mg protein specific activity and 81.2% yield. Subsequently, gel filtration chromatography further increased the specific activity to 253 ± 0.11 units/mg protein with 4.96-fold purification and 66.3% yield. The β-galactosidase in this study was successfully obtained with few purification steps from a low-cost production condition, thus being valuable for the industry in terms of economic point of view. Moreover, the purified β-galactosidase from *E. coli* was analyzed by SDS-PAGE (Fig. 2), which reveals a single band with a molecular mass of 72 kDa. It is worth noting that the molecular mass of a protein band around 72 kDa and sequences of the PCR amplicons submitted to GenBank are of 1577 bp (493 amino acids residues) corresponding to a partial coding sequence of LacZ gene. A modification occurred after UV exposure that could explain the differences and the gain observed in the β-galactosidase productivity. In agreement with our results [38], the recombinant β-galactosidase from *Bacillus licheniformis* was purified by a single-step purification protocol using a Ni-Sepharose 6 fast-flow column, and the purified enzyme shows a molecular mass of 75 kDa when analyzed by SDS-PAGE. Consistently, [16] also purified the recombinant β-galactosidase from *Bacillus subtilis* with a Superdex G-200 column step and the purified recombinant enzyme was exhibiting a single-protein band with an apparent molecular mass of 75 kDa, in agreement with the theoretical molecular weight of 75,164.0 kDa calculated for the YesZ amino acid sequence involving the C-terminal extension [39]. cloned the β-galactosidase of *Thermotoganaph thophila* and expressed it in *E. coli*, and the SDS-PAEG of the purified recombinant enzyme exhibited a molecular weight of 70 kDa.

**Table 3:** Purification scheme for β-galactosidase from *E. coli* M-KH-UV-Tra44

Purification step	Total protein (mg/ml)	Total activity (U/ml)	Specific activity (U/mg)	Yield (%)	Purification fold
Crude enzyme	4.86 ± 0.17	251.7 ± 1.2	51.7 ± 1.3	100	1
Ammonium sulfate (60–80%)	1.28 ± 0.14	204 ± 1.6	159.3 ± 0.81	81.2	3.07
Sephadex G-100	0.66 ± 0.11	167 ± 0.82	253 ± 0.11	66.3	4.96

### Effect of pH, temperature, and salinity on β-galactosidase

**Fig. 3:**
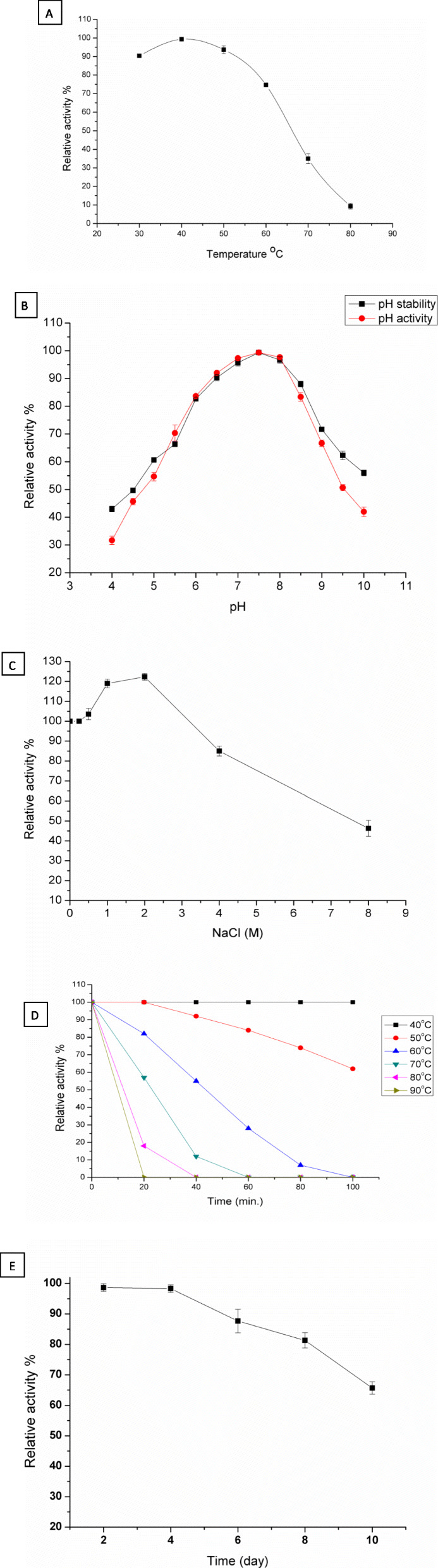
Biochemical characterizations of the trans-mutant *E. coli* DH5α LacZ. **a** Effect of temperatures (30–80 °C) on the enzyme activity carried out at pH 6.5 and 5 mM of *o*NPG. **b** Effect of pH on the enzyme activity and stability using different buffers (pH 4–10) at 40 °C and 5 mM of *o*NPG. **c** Effect of the ionic strength on the enzyme activity using NaCl (0.25–8 M) at 40 °C, pH 7.5 and 5 mM *o*NPG. **d** Thermal stability of the enzyme carried out at different temperatures (40–90 °C). **e** Enzyme stability at 4 °C, pH 7.5 and 5 mM *o*NPG

All experiments were investigated with *o*NPG as the substrate. The effect of temperature on β-galactosidase activity was assessed under standard assay conditions (pH 6.5, for 10 min) except that the reaction temperatures were adjusted between 20 and 80 °C. The maximal catalytic activity was obtained at 40 °C; meanwhile, a mild decrease in the enzymatic activity was noticed by increasing the reaction temperature above 50 °C (Fig. 3a). On the other hand, the effect of pH on β-galactosidase activity was investigated under standard assay conditions (40 °C, for 10 min), but the reaction pH was adjusted between 4.0 and 10.0 by different buffering systems. The maximal catalytic activity was found to be near the neutral pH in the 6.0–8.0 pH range, while somewhat influencing the enzymatic activity noted in the pH range of 9–10 (Fig. 3b). Due to a potential industrial applicability of β-galactosidase, determination of the enzymatic activity was carried out under ionic strength conditions (0–8 M NaCl). The ionic strength was found to notably increase the enzymatic activity even at 2.0 M, while further increases in salinity (4-8 M) caused reduction in the relative activity as shown in Fig. 3c. Incubation of β-galactosidase with different temperatures (4–80 °C) at pH 6.5 without substrate was also analyzed to evaluate the thermal stability of the enzyme. The enzyme was quite stable below 40 °C, but the enzymatic activity was decreased to 85% and 30% when kept at 50 °C and 60 °C, respectively, for 1 h (Fig. 3d). Moreover, the enzyme completely lost its initial activity after incubation at 70 °C for 1 h. The residual activity of lyophilized enzyme after being kept at 4 °C for 8 days was about 81.3% which suggests that enzyme preparation is suitable for lactose hydrolysis in milk (Fig. 3e). Regarding the pH stability, the enzyme showed remarkable structural stability over a wide range of pH (6.0–8.5). The enzyme had remained more than 90% of its original activity when incubated in the pH range 6.5-8.0 for 24 h and then moderately decreased at alkaline pH region (9.0–10.0) (Fig. 3b).

In accordance with our results, [38] reported that the β-galactosidase LacA from *Bacillus licheniformis* DSM13, which was cloned and expressed in *E. coli*TOP10, was proved to have a maximum pH activity at 6.5, and the enzyme was stable in the pH range of 5 to 8, with an optimum temperature of 50 °C and thermal stability under 40 °C [35]. design the recombinant β-galactosidase gene (PbBGal2A) from *Paenibacillus barengoltzii* expressed in *E. coli*; the enzyme displayed an optimal activity at pH 7.5 with ionic stability over the pH range of 6.0-8.0, and the highest enzymatic activity was demonstrated at 45 °C. Interestingly, β-galactosidase possessed a sufficient stability at 4 °C for up to 72 h (Fig. 3d) which validates its application in lactose-free milk process due to the hydrolysis of lactose in milk carried out at low temperature (under 8 °C), and after that, the enzyme was killed when exposed to temperature above 65 °C. In addition, the thermal stability of β-galactosidase up to 50 °C emphasizes the potential utilization in lactose conversion in different dairy industrial processes, in which most of *Lactobacillia* and *E. coli* β-galactosidases can be used only at 37 °C [37]

### Effect of various cations on β-galactosidase catalytic activity

The effect of various cations (chloride salt) and chemical compounds (carbonyl reagents, thiol reagents, chelating agent, or other chemicals) was evaluated at the standard assay condition (Table 4). Among mono-, di-, and tri-valent metal ions, the enzymatic activity was enhanced with 10 mM of Mg^+2^ and Mn^+2^. For its optimal activity, different concentrations of these di-valent cations were investigated which indicated that the enzyme activity requires 5.0 mM Mn^2+^and 2.5 mM Mg^2+^ to increase to 132% and 126%, respectively (data not shown). This activation of enzyme may be due to the interaction of β-galactosidase with di-valent ions. The enzyme activity was inhibited strongly (more than 50%) in the presence of Ba^+2^, Li^+2^, Cu^+2^, Co^+2^, or Fe^+3^ and slightly by Zn^+2^, Cr^+3^, or Ni^+2^, suggesting an interaction with the active site of the enzyme, while the enzyme activity did not alter with Na^+^, K^+^, Ca^+2^, or Cd^+2^. On the other hand, the enzyme activity was significantly inhibited by the presence of each of EDTA and SDS and slightly affected by sodium azide, 8-hydroxyquinoline, β-mercabtoethanol, or hydroxylamine. Thus, the carbonyl and sulfhyryl group are not concerned in the enzyme activity. Surprisingly, the inhibition of enzymatic activity by EDTA was clearly recovered in the presence of 1.0 mM Mn^2+^ (data not shown). These results clearly indicate that the enzyme having a metallic nature which Mn^2+^play an important role for activating and protecting the active site of the enzyme against inhibitors [3]. Similarly, the recombinant β-galactosidase gene (PbBGal2A) from *Paenibacillus barengoltzii* CAU904 was reported as a metal-dependent enzyme [35], which was strongly inactivated by EDTA and highly stimulated by the presence of each of K^+^, Na^+^,Mn^2+^, and Mg^2+^. Also, [38] shows that the presence of Na^+^ or Mn^+2^ could enhance the enzyme activity, but when their synergistic effect together was tested, there were no any stimulation. It is worth mentioning that Ca^+2^ and Zn^+2^ were known as an inhibitor of some β-galactosidase [16, 35, 40]. However, Ca^+2^ did not display any change in the enzymatic activity even at 10 mM while a slightly decrease in the activity was noticed at 10 mM of Zn^+2^. These findings were very important in the lactose hydrolysis process in milk or whey which was containing high level of free Ca^2+^in solution [38].

**Table 4:** Effect of metal ions and additives on β-galactosidase activity

Metal ions (chloride salt, 10 mM)	Relative activity (%)	Additives (1 mM)	Relative activity (%)
Na^+^	98 ± 1.5	Sodium azide	96 ± 1
K^+^	100 ± 0.88	8-Hydroxyquinoline	91.3 ± 1.5
Mg^+2^	122 ± 1	EDTA	55.3 ± 1.1
Mn^+2^	116 ± 1.7	SDS	62 ± 1
Zn^+2^	83 ± 2.1	DTT	100 ± 0.88
Ca^+2^	97 ± 1.1	β-Mercabtoethanol	82.5 ± 1.3
Ba^+2^	42 ± 1.5	Hydroxylamine	93 ± 0.87
Li^+2^	45.7 ± 1.3		
Cu^+2^	8.2 ± 1		
Co^+2^	39.5 ± 1.6		
Cd^+2^	98 ± 2.9		
Cr^+3^	82 ± 0.84		
Ni^+2^	74.9 ± 1.6		
Fe^+3^	22.9 ± 1.4		

### Effect of organic solvents and saccharides on β-galactosidase

**Fig. 4:**
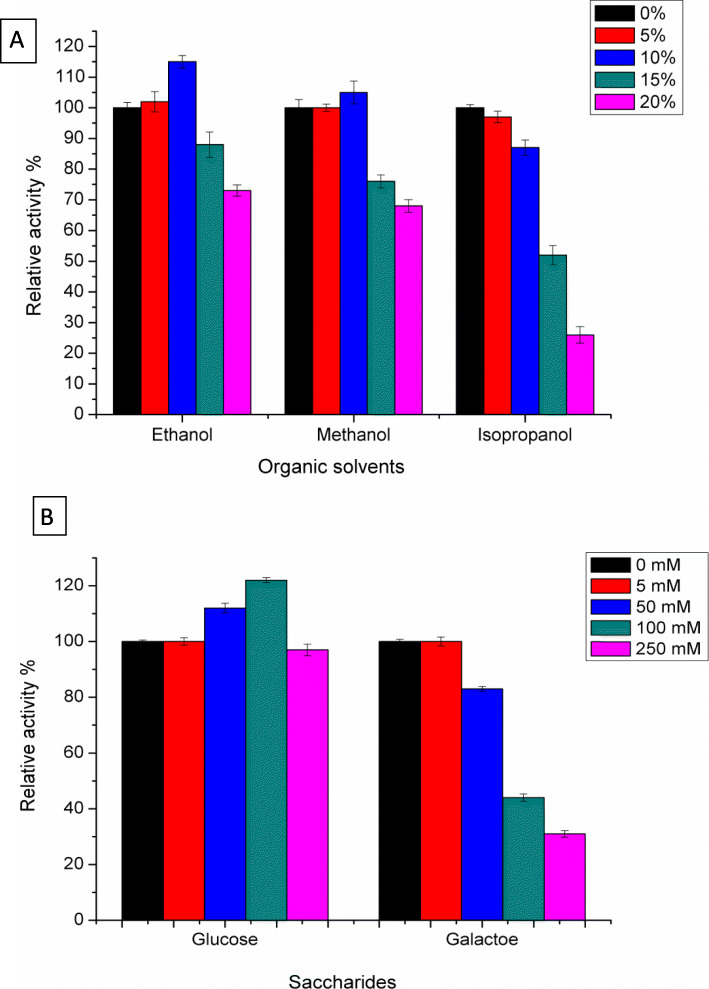
Enzymatic activity influence in the presence of each of **a** organic solvents (0–20%) and **b** saccharides (0–250 mM), carried out at 40 °C, pH 7.5 and 5 mM of *o*NPG

Evaluation of the effect of organic solvents is playing an important role in the industrial applications of β-galactosidase [36]. As can be seen in (Fig. 4a), the effect of different concentrations of ethanol, methanol, and isopropanol (0–20%) on the enzymatic activity was investigated. The enzymatic activity was potentiated and increased up to 15 and 5% of its initial activity in the presence of 10% ethanol or methanol, respectively. However, at a concentration of 15% of ethanol and methanol, β-galactosidase kept a level of enzymatic activity similar to the control. A dramatical decrease of the β-galactosidase activity was found to be correlated with an increased concentration of ethanol or methanol above 15%, and when the enzyme was assayed with 20% of ethanol or methanol, a somewhat reduction in activity (approximately 30–35%) was noticed. Conversely, the enzyme did not had any effect on the activity even at 5%, while it was significantly inhibited in the presence of isopropanol at 10% and 20% concentrations. Incubation of β-galactosidase with methanol, ethanol, or isopropanol at 10 and 20% (v/v for 2 h) was also investigated in order to determine the solvent stability (data not shown). The enzyme retained its catalytic activity when incubated with each of ethanol and methanol at 10% for 1 h, while there was a slight decrease in the enzymatic activity in the presence of isopropanol for the same period. Reduction in enzymatic activity reached to 13%, 27%, and 41% after a 2-h incubation with 20% of ethanol, methanol, and isopropanol, respectively. The organic solvent stability of *E. coli* DH5α β-galactosidase makes it an excellent candidate for utilization in biotechnological sectors. Earlier, organic solvent stability of *Halorubumlacus profundi* β-galactosidase candidate it to applied in galacto-oligosaccharides synthesis from lactose [36, 41]. For instance, synthesis of *N*-acetyl-lactosamine by *Bacillus circulans* β-galactosidase required a tert-butanol-water mixture, which reflects the high benefits of enzymatic stability in organic solvents [42]. On the other hand, the *E. coli* β-galactosidase activity could be increased in the presence of ethanol or methanol at 10% which may be resulting in the galactosyl transferase activity, making ethanol or methanol as preferred acceptor of glycosyl residues during enzymatic reaction [43].

In addition to organic solvents, different concentrations of glucose or galactose were added to the reaction assay (using *o*NPG as a substrate) to determine the behavior of β-galactosidase in the presence of these inhibitors (Fig. 4b). Obviously, the enzyme activity was sharply inhibited in the presence of galactose, retaining approximately 44% of its initial activity when the reaction assay was performed with 100 mM of galactose. In contrast, the presence of glucose in the reaction mixture was found as an activator at 100 mM in which the enzyme activity increased with 122% of its initial activity. Our results were consistent with the results demonstrated in earlier studies [2, 35, 38]. Indeed, a lot of microbial β-galactosidases are inhibited by d-galactose, which exhibits a severe problem that reduces the utilization of β-galactosidase in industrial sectors [2]. Meanwhile, the presence of glucose was found to be a minor promoter of enzyme activity in some reports [35, 37]. Reduction in enzymatic activity in the presence of high-galactose concentrations may be attributed to the interference with substrate binding to the enzyme active site directly or indirectly, lowering the reaction rate [2]. It is interesting that the incompetence of *E. coli* β-galactosidase was completely recovered when the substrate was increased to the same galactose concentration in the reaction mixture, explaining that the interfering of the enzyme with d-galactose is competitive (data not shown). Furthermore, D-galactose competitively inhibited both the hydrolysis of lactose and *o*NPG. Increasing substrate concentration can recover enzymatic reduction by competitive inhibition, in which the inhibitor and the substrate compete for the same active binding site of the enzyme. Therefore, increasing the substrate concentration to equal or greater values than those of the inhibitor favors the binding of the enzyme to the substrate, which is reflected in the reversibility of enzymatic inhibition [43]. The reversibility of inhibition by d-galactose and stability to organic solvents ensures the potential application of this enzyme for their ability to work in the industrial sector [2, 36].

### Substrate specificity and kinetic studies of β-galactosidase

Specificity of the *E. coli* β-galactosidase was determined by a hydrolysis reaction assay including 5 mM of chromogenic substrates (*o*NPG or pNPG) and 100 mM of natural substrates (Lactose; Raffinose; Xylose or Starch). The enzyme displayed higher activity toward the chromogenic (*o*NPG) and natural substrates (lactose). In contrast, the enzyme demonstrated little activity in case of p-nitrophenyl-d-galactopyranoside or no activity in case of other saccharides (data not shown). Therefore, β-galactosidase kinetic constants calculated for the hydrolysis of lactose and *o*NPG under standard assay reactions are presented in Table 5. Kinetic parameters revealed that the trans-mutant *E. coli* β-galactosidase had a high affinity of 1.4 mM, 14.2 U/mg/min, for *o*NPG followed by 12.92 mM, 6.4 U/mg/min, for lactose, while the enzyme exhibits a *K*_cat_ value of 312 S^−1^ and 93S^−1^ for *o*NPG and lactose, respectively. As shown in Table 5, catalytic coefficient (*K*_cat_*/K*_m_) was calculated as 219.7  S^−1^ mM^−1^ for *o*NPG and 7.1  S^−1^ mM^−1^ for lactose, suggesting that binding of one ligand molecule to the active site decreases the affinity for ligand binding to other protein subunits in the enzymatic structure [2]. The increase in substrate concentration here increased the β-galactosidase activity, after which it is saturated assuming the competitive inhibition of the end product (d-galactose). The smaller *K*_m_ value indicates the high affinity and efficient catalytic role of the enzyme against the substrate [44]. In fact, the catalytic coefficient (*K*_cat_*/K*_*m*_) for both, lactose and *o*NPG, indicates that *o*NPG is clearly the preferred substrate, because of more favorable *K*_m_ and *K*_cat_ values. It is interestingly that *E. coli* β-galactosidase was close to those showed for other already reported in the literature [35]. reported for *Paenibacillus* β-galactosidase high affinity toward the *o*NPG with *K*_m_ of 1.13 mM and low affinity for lactose with *K*_m_ of 43.2 mM [31]. founded a high affinity of *Lactococcus lactis* IL1403 β-galactosidases toward both substrates of 0.12 mM and 0.82 Mm for oNPG and lactose, respectively. Likewise, the catalytic efficiency of *E. coli* B-gal toward lactose is proved to be higher than those studied from *Paenibacillus* [35], *Bifidobacterium breve* DSM 20213, *Lactobacillus delbrueckii* subsp. *Bulgaricus* DSM20081 [40], and *B. licheniformis* [38].

**Table 5:** Enzyme kinetics for the selected substrates

Substrate	*V*_max_ (U/mg/min)	*K*_m_ (mM)	*K*_cat_ (S^−1^)	*K*_cat_*/K*_m_ (1/mM S)
*o*NPG	14.2	1.4	312	219.7
Lactose	6.4	12.92	93	7.1

Enzyme activity for the selected substrates was assessed under standard conditions using 0.5–25 mM of O-nitrophenyl b-d-galactopyranoside or 20–360 mM of lactose in 50 mM sodium phosphate buffer (pH 7.8) at 40 °C for 10 min. For lactose-hydrolyzing activity, glucose oxidase was used to determine the specific activity. The *K*_m_ and *V*_max_ values were calculated by a Lineweaver-Burk plot

### Lactose bioconversion by β-galactosidase

**Fig. 5:**
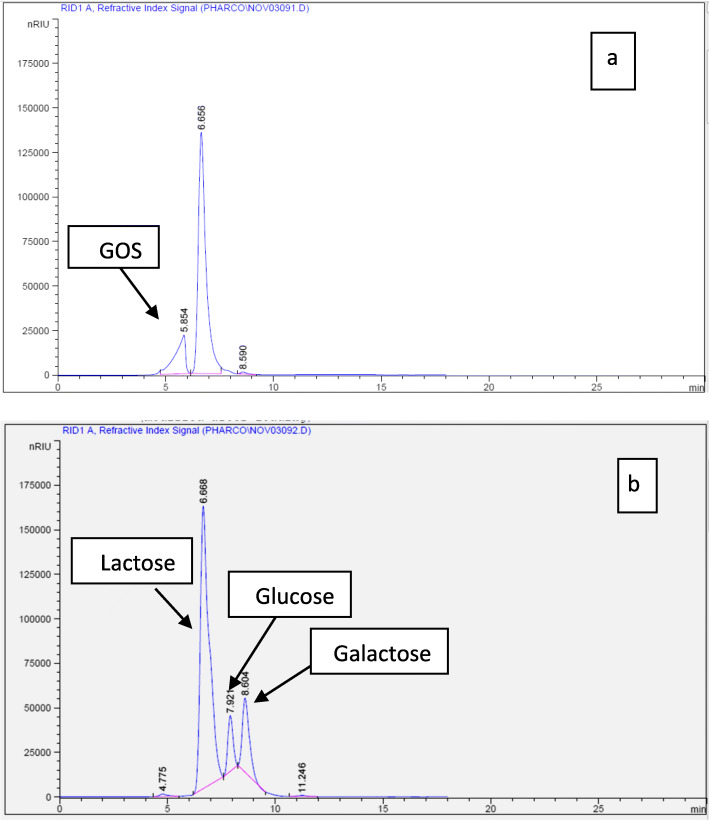
HPLC Profile of the end product resulting from hydrolysis of d-lactose with β-galactosidase. **a** After 12 h of reaction. **b** After 24 h of reaction

Hydrolysis of lactose via β-galactosidase-catalyzed conversion is of interest in dairy industry due to the production of lactose-free milk as well as for the formation of galacto-oligosaccharide (GOS) [2]. The bioconversion of lactose across LacZ was proved using HPLC analysis. A time course profile of GOS and monosaccharide synthesis using 50 g/L at pH 7.5 and 37 °C, and employing 12 U of β-galactosidase/ml was monitored. Results displayed that 5% (w/v) of the lactose was efficiently hydrolyzed with 33% to yield mainly galactose and glucose after 24 h (Fig. 5a, b). However, GOS formation could be detected with low levels, and the maximum yield of 13% (w/w) was found after 12 h. Around 40% of lactose was converted within 24 h of the reaction at 37 °C and reflects that the reaction slowed down (Fig. 5b), which may be correlated with reaction temperature [38]. found that higher temperatures in reaction increases GOS yield, since an increase in temperature contributes to the improved solubility of lactose, which is relatively low at room temperatures. Interestingly, the continuous hydrolysis of lactose at the ambient temperatures in addition to the processing not inhibited by the generated product (i.e., galactose) may enable the trans-mutant *E. coli* DH5α β-galactosidase to become a great candidate in lactose-free milk industries. In other words, the lower GOS formation during the reaction might be related to its relatively high *K*_m_ and high binding power to glucose moiety [37]. Therefore, LacZ may be appropriate for the lactose bioconversion in industry at higher temperatures. Our results is lower than those reported by other β-galactosidases from *Bacillus circulans* [45] and *Paenibacillus barengoltzii* CAU904 [35] and higher than those of the β-galactosidases from *Bacillus licheniformis* DSM 13 [38] which possess GOS yields of 48.3%, 47.9%, and 12%, respectively.

## Conclusion

Bacterial trans-conjugation was used as a tool to activate β-galactosidase productivity through alpha complementation between pGEM harboring *E. coli* LK111 and recipient *E. coli* DH5α (∆M15lacZ). Active trans-conjugant isolates further improved its productivity through UV mutagenesis, and M-KH-UV-Tran44 trans-mutant was proved to be the best producer with 251.7 U/ml among the most potent six trans-mutant isolates. β-Galactosidase with molecular mass of 72 kDa was successfully purified by 4.96-fold with 66.3% yield using ammonium sulfate precipitation and gel filtration chromatography. Overall, the purified enzyme was found to be having a high activity near neutral pH (6–8) and high activity in salt concentrations up to 2 M, and the efficient hydrolysis of lactose at ambient temperatures suggests suitability for storage conditions of dairy products. Furthermore, the purified enzyme was employed to bioconvert lactose, and attractive hydrolysis rates were obtained at low temperature. The unique properties of the *E. coli* KH-UV-Tran44-β-galactosidase may make it a great candidate for application in different food industries.

## Supplementary Information

**Additional file 1.** Supplementary data.

## Data Availability

The authors declare that all generated and analyzed data are included in the article. All bacterial species (different *E. coli* models) were kindly obtained from Applied Microbial Genetics Lab, Cytology and Genetics Dept., National Research Centre (NRC), Dokki, Egypt.

## Abbreviations

PCR: Polymerase chain reaction SDS-PAGE Sodium dodecyl sulfate-polyacrylamid gel electrophoresis UV Ultraviolet HGT Horizontal gel transfer Rif Rifampicin Kn Kanamycin LB Laura Bertani *o*NPG Ortho-nitrophenyl-β-galactoside BSA Bovine serum albumin *V*_max_ Maximum reaction velocity *K*_m_ Michaelis-Menten constant *K*_cat_ Turn over number *K*_cat_/*K*_m_ Catalytic coefficient HPLC High-performance liquid chromatography ± SD ± Standard division CFU Colony-forming unit MEGA Molecular Evolutionary Genetics Analysis EDTA Ethylene diamine tetra-acetic acid GOS Galacto-oligosaccharide
