# Supplementary material for: Activation of LacZ gene in Escherichia coli DH5α via α-complementation mechanism for β-galactosidase production and its biochemical characterizations
Source: J Genet Eng Biotechnol. 2020 Dec 2;18:80. doi: 10.1186/s43141-020-00096-w (PMC7710787; doi:10.1186/s43141-020-00096-w)
Supplement: Supplementary file 1 — Additional file 1. Supplementary data. [file 43141_2020_96_MOESM1_ESM.docx]

**SUPPLEMENTARY MATERIALS**


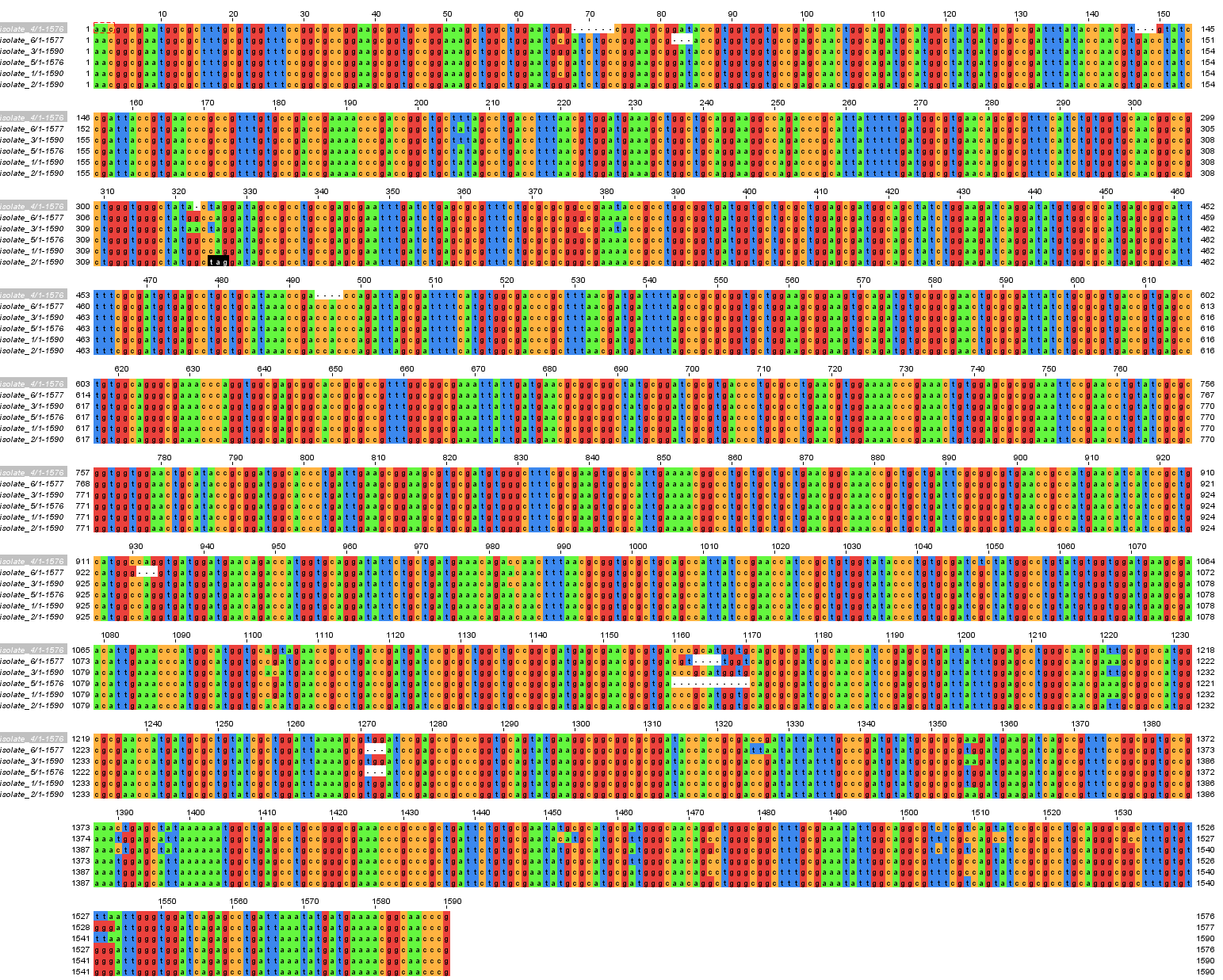


**Supplementary data Fig. S1 LacZ deoxyribonucleotide sequence alignments of six beta-galactosidase producing trans-mutant *E.coli* DH5α obtained from this study, in this alignment; Minimum Sequence Length was 1576, Maximum Sequence Length was 1590, and average Length was 1583.**


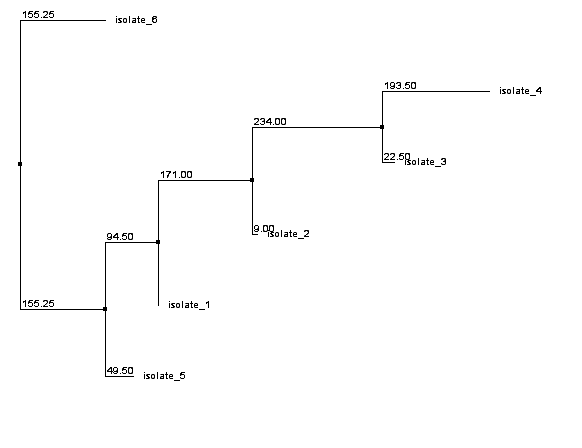


**Supplementary data Fig S2 Neighboring joining DNA Tree depends on LacZ sequence of the tested trans-mutant isolates**


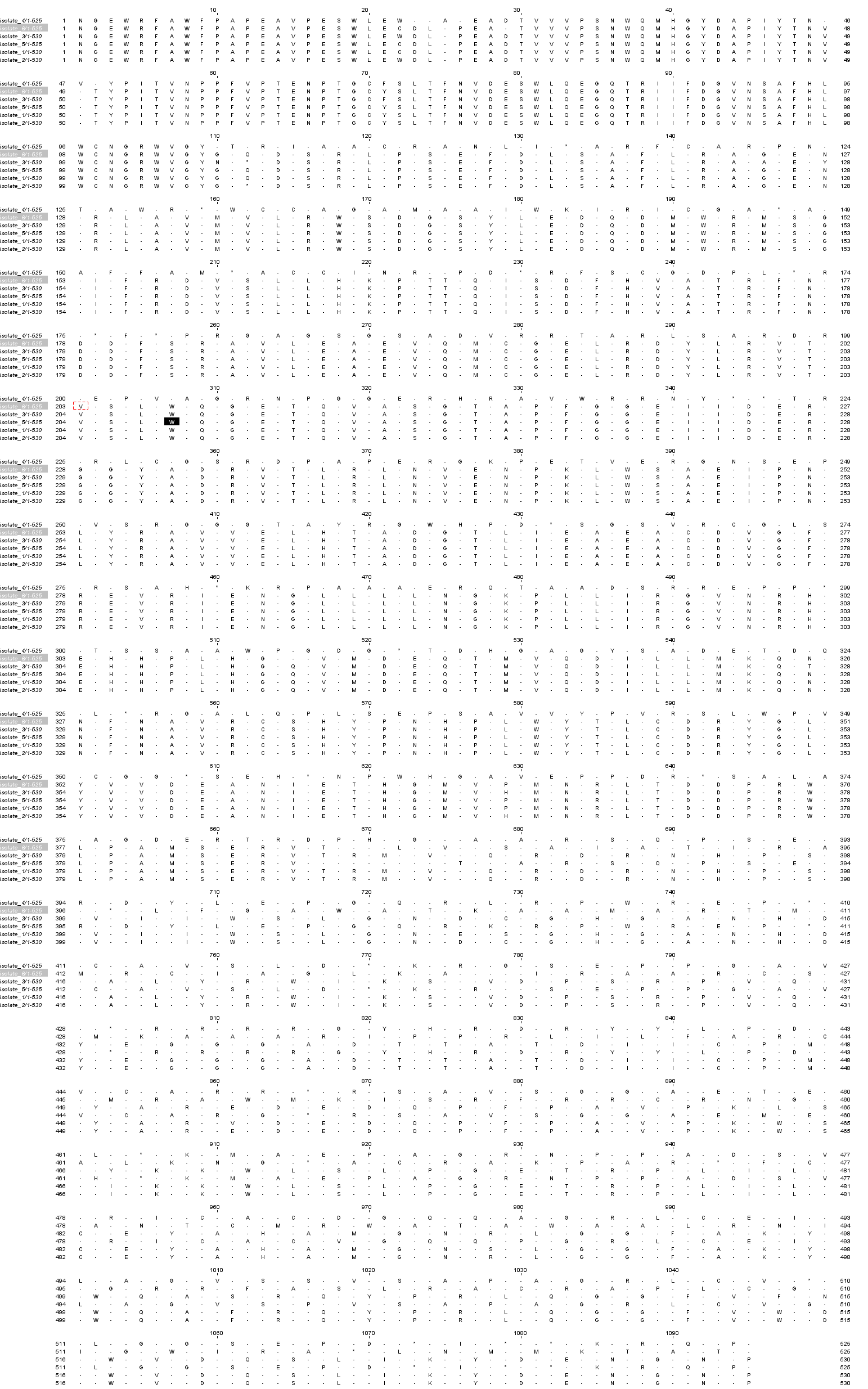
 **Supplementary data Fig. S3 protein sequence obtained from LacZ sequence of tested trans-mutant isolates through Jalview software 2.11.1.**

**Supplementary data Table S1 LacZ gene sequence of the six UV-mutants:**

| **Mutants**  **code** | **GenBank accession**  **Number** | **LacZ gene sequence** | **Molecular size (bps)** |
| --- | --- | --- | --- |
| MKUV-Tra2 | MN172239 | acggcgaatggcgctttgcgtggtttccggcgccggaagcggtgccggaaagctggctggaatgcgatctgccggaagcggataccgtggtggtgccgagc  aactggcagatgcatggctatgatgcgccgatttataccaacgtgacctatccgattaccgtgaacccgccgtttgtgccgaccgaaaacccgaccggctgctata  gcctgacctttaacgtggatgaaagctggctgcaggaaggccagacccgcattatttttgatggcgtgaacagcgcgtttcatctgtggtgcaacggccgctgggt  gggctatggccaggatagccgcctgccgagcgaatttgatctgagcgcgtttctgcgcgcgggcgaaaaccgcctggcggtgatggtgctgcgctggagcgat  ggcagctatctggaagatcaggatatgtggcgcatgagcggcatttttcgcgatgtgagcctgctgcataaaccgaccacccagattagcgattttcatgtggcgac  ccgctttaacgatgattttagccgcgcggtgctggaagcggaagtgcagatgtgcggcgaactgcgcgattatctgcgcgtgaccgtgagcctgtggcagggcga  aacccaggtggcgagcggcaccgcgccgtttggcggcgaaattattgatgaacgcggcggctatgcggatcgcgtgaccctgcgcctgaacgtggaaaacccg  aaactgtggagcgcggaaattccgaacctgtatcgcgcggtggtggaactgcataccgcggatggcaccctgattgaagcggaagcgtgcgatgtgggctttcgc  gaagtgcgcattgaaaacggcctgctgctgctgaacggcaaaccgctgctgattcgcggcgtgaaccgccatgaacatcatccgctgcatggccaggtgatggatg  aacagaccatggtgcaggatattctgctgatgaaacagaacaactttaacgcggtgcgctgcagccattatccgaaccatccgctgtgtataccctgtgcgatcgctatg  gcctgtatgtggtggatgaagcgaacattgaaacccatggcatggtgccgatgaaccgcctgaccgatgatccgcgctggctgccggcgatgagcgaacgcgtgac  ccgcatggtgcagcgcgatcgcaaccatccgagcgtgattatttggagcctgggcaacgaaagcggccatggcgcgaaccatgatgcgctgtatcgctggattaaaa  gcgtggatccgagccgcccggtgcagtatgaaggcggcggcgcggataccaccgcgaccgatattatttgcccgatgtatgcgcgcgtggatgaagatcagccgtttc  cggcggtgccgaaatggagcattaaaaaatggctgagcctgccgggcgaaacccgcccgctgattctgtgcgaatatgcgcatgcgatgggcaacagcctgggcggc  tttgcgaaatattggcaggcgtttcgccagtatccgcgcctgcagggcggctttgtgtgggattgggtggatcagagcctgattaaatatgatgaaaacggcaacccg | 1590 |
| MKUV-Tra10 | MN172240 | aacggcgaatggcgctttgcgtggtttccggcgccggaagcggtgccggaaagctggctggaatgggatctgccggaagcggataccgtggtggtgccgagcaact  ggcagatgcatggctatgatgcgccgatttataccaacgtgacctatccgattaccgtgaacccgccgtttgtgccgaccgaaaacccgaccggctgctatagcctgacct  ttaacgtggatgaaagctggctgcaggaaggccagacccgcattatttttgatggcgtgaacagcgcgtttcatctgtggtgcaacggccgctgggtgggctatggctagga  tagccgcctgccgagcgaatttgatctgagcgcgtttctgcgcgcgggcgaaaaccgcctggcggtgatggtgctgcgctggagcgatggcagctatctggaagatcagg  atatgtggcgcatgagcggcatttttcgcgatgtgagcctgctgcataaaccgaccacccagattagcgattttcatgtggcgacccgctttaacgatgattttagccgcgcggtg  ctggaagcggaagtgcagatgtgcggcgaactgcgcgattatctgcgcgtgaccgtgagcctgtggcagggcgaaacccaggtggcgagcggcaccgcgccgtttggcgg  cgaaattattgatgaacgcggcggctatgcggatcgcgtgaccctgcgcctgaacgtggaaaacccgaaactgtggagcgcggaaattccgaacctgtatcgcgcggtggtgg  aactgcataccgcggatggcaccctgattgaagcggaagcgtgcgatgtgggctttcgcgaagtgcgcattgaaaacggcctgctgctgctgaacggcaaaccgctgctgattc  gcggcgtgaaccgccatgaacatcatccgctgcatggccaggtgatggatgaacagaccatggtgcaggatattctgctgatgaaacagaacaactttaacgcggtgcgctgcag  ccattatccgaaccatccgctgtggtataccctgtgcgatcgctatggcctgtatgtggtggatgaagcgaacattgaaacccatggcatggtgcacatgaaccgcctgaccgatgat  ccgcgctggctgccggcgatgagcgaacgcgtgacccgcatggtgcagcgcgatcgcaaccatccgagcgtgattatttggagcctgggcaacgattgcggccatggcgcgaac  catgatgcgctgtatcgctggattaaaagcgtggatccgagccgcccggtgcagtatgaaggcggcggcgcggataccaccgcgaccgatattatttgcccgatgtatgcgcgcgaa  gatgaagatcagccgtttccggcggtgccgaaatggagcattaaaaaatggctgagcctgccgggcgaaacccgcccgctgattctgtgcgaatatgcgcatgcgatgggcaacaggc  tgggcggctttgcgaaatattggcaggcgtttcgtcagtatccgcgcctgcagggcggctttgtgtgggattgggtggatcagagcctgattaaatatgatgaaaacggcaacccg | 1590 |
| MKUV-Tra25 | MN172241 | aacggcgaatggcgctttgcgtggtttccggcgccggaagcggtgccggaaagctggctggaatgggatctgccggaagcggataccgtggtggtgccgagcaactggcagatgc  atggctatgatgcgccgatttataccaacgtgacctatccgattaccgtgaacccgccgtttgtgccgaccgaaaacccgaccggctgctttagcctgacctttaacgtggatgaaagctg  gctgcaggaaggccagacccgcattatttttgatggcgtgaacagcgcgtttcatctgtggtgcaacggccgctgggtgggctataactaggatagccgcctgccgagcgaatttgatct  gagcgcgtttctgcgcgcggccgaataccgcctggcggtgatggtgctgcgctggagcgatggcagctatctggaagatcaggatatgtggcgcatgagcggcatttttcgcgatgtga  gcctgctgcataaaccgaccacccagattagcgattttcatgtggcgacccgctttaacgatgattttagccgcgcggtgctggaagcggaagtgcagatgtgcggcgaactgcgc  gattatctgcgcgtgaccgtgagcctgtggcagggcgaaacccaggtggcgagcggcaccgcgccgtttggcggcgaaattattgatgaacgcggcggctatgcggatcgcgtgaccc  tgcgcctgaacgtggaaaacccgaaactgtggagcgcggaaattccgaacctgtatcgcgcggtggtggaactgcataccgcggatggcaccctgattgaagcggaagcgtgcgatgtg  ggctttcgcgaagtgcgcattgaaaacggcctgctgctgctgaacggcaaaccgctgctgattcgcggcgtgaaccgccatgaacatcatccgctgcatggccaggtgatggatga  acagaccatggtgcaggatattctgctgatgaaacagaccaactttaacgcggtgcgctgcagccattatccgaaccatccgctgtggtataccctgtgcgatcgctatggcctgtatgtg  gtggatgaagcgaacattgaaacccatggcatggtgcacatgaaccgcctgaccgatgatccgcgctggctgccggcgatgagcgaacgcgtgacccgcatggtgcagcgcgatcgca  accatccgagcgtgattatttggagcctgggcaacgattgcggccatggcgcgaaccatgatgcgctgtatcgctggattaaaagcgtggatccgagccgcccggtgcagtatgaaggcgg  cggcgcggataccaccgcgaccgatattatttgcccgatgtatgcgcgcgaagatgaagatcagccgtttccggcggtgccgaaactgagctataaaaaatggctgagcctgccgggcgaa  acccgcccgctgattctgtgcgaatatgcgcatgcgatgggcaacaggctgggcggctttgcgaaatattggcaggcgtctcgtcagtatccgcgcctgcagggcggctttgtgtttaattgggt  ggatcagagcctgattaaatatgatgaaaacggcaacccg | 1590 |
| MKUV-Tra35 | MN172242 | aacggcgaatggcgctttgcgtggtttccggcgccggaagcggtgccggaaagctggctggaatgggcggaagcggataccgtggtggtgccgagcaactggcagatgcatgg  ctatgatgcgccgatttataccaacgtgtatccgattaccgtgaacccgccgtttgtgccgaccgaaaacccgaccggctgctttagcctgacctttaacgtggatgaaagctggctgca  ggaaggccagacccgcattatttttgatggcgtgaacagcgcgtttcatctgtggtgcaacggccgctgggtgggctatactaggatagccgcctgccgagcgaatttgatctgagcgc  gtttctgcgcgcggccgaataccgcctggcggtgatggtgctgcgctggagcgatggcagctatctggaagatcaggatatgtggcgcatgagcggcatttttcgcgatgtgagcctgct  gcataaaccgaccagattagcgattttcatgtggcgacccgctttaacgatgattttagccgcgcggtgctggaagcggaagtgcagatgtgcggcgaactgcgcgattatctgcgcgtgac  cgtgagcctgtggcagggcgaaacccaggtggcgagcggcaccgcgccgtttggcggcgaaattattgatgaacgcggcggctatgcggatcgcgtgaccctgcgcctgaacgtggaaa  acccgaaactgtggagcgcggaaattccgaacctgtatcgcgcggtggtggaactgcataccgcggatggcaccctgattgaagcggaagcgtgcgatgtgggctttcgcgaagtgcgcatt  gaaaacggcctgctgctgctgaacggcaaaccgctgctgattcgcggcgtgaaccgccatgaacatcatccgctgcatggccaggtgatggatgaacagaccatggtgcaggatattctgctga  tgaaacagaccaactttaacgcggtgcgctgcagccattatccgaaccatccgctgtggtataccctgtgcgatctctatggcctgtatgtggtggatgaagcgaacattgaaacccatggcatggtg  cagtagaaccgcctgaccgatgatccgcgctggctgccggcgatgagcgaacgcgtgacccgcatggtgcagcgcgatcgcaaccatccgagcgtgattatttggagcctgggcaacgattgcg  gccatggcgcgaaccatgatgcgctgtatcgctggattaaaagcgtggatccgagccgcccggtgcagtatgaaggcggcggcgcggataccaccgcgaccgatattatttgcccgatgtatgcgc  gcgaagatgaagatcagccgtttccggcggtgccgaaactgagctataaaaaatggctgagcctgccgggcgaaacccgcccgctgattctgtgcgaatatgcgcatgcgatgggcaacaggctgg  gcggctttgcgaaatattggcaggcgtctcgtcagtatccgcgcctgcagggcggctttgtgtttaattgggtggatcagagcctgattaaatatgatgaaaacggcaacccg | 1576 |
| MKUV-Tra44 | MN172243 | aacggcgaatggcgctttgcgtggtttccggcgccggaagcggtgccggaaagctggctggaatgcgatctgc  cggaagcggataccgtggtggtgccgagcaactggcagatgcatggctatgatgcgccgatttataccaacgtga  cctatccgattaccgtgaacccgccgtttgtgccgaccgaaaacccgaccggctgctatagcctgacctttaacgtgg  atgaaagctggctgcaggaaggccagacccgcattatttttgatggcgtgaacagcgcgtttcatctgtggtgcaacg  gccgctgggtgggctatggccaggatagccgcctgccgagcgaatttgatctgagcgcgtttctgcgcgcgggcgaa  aaccgcctggcggtgatggtgctgcgctggagcgatggcagctatctggaagatcaggatatgtggcgcatgagcggc  atttttcgcgatgtgagcctgctgcataaaccgaccacccagattagcgattttcatgtggcgacccgctttaacgatgatttt  agccgcgcggtgctggaagcggaagtgcagatgtgcggcgaactgcgcgattatctgcgcgtgaccgtgagcctgtgg  cagggcgaaacccaggtggcgagcggcaccgcgccgtttggcggcgaaattattgatgaacgcggcggctatgcggatc  gcgtgaccctgcgcctgaacgtggaaaacccgaaactgtggagcgcggaaattccgaacctgtatcgcgcggtggtggaa  ctgcataccgcggatggcaccctgattgaagcggaagcgtgcgatgtgggctttcgcgaagtgcgcattgaaaacggcctgc  tgctgctgaacggcaaaccgctgctgattcgcggcgtgaaccgccatgaacatcatccgctgcatggccaggtgatggatgaa  cagaccatggtgcaggatattctgctgatgaaacagaacaactttaacgcggtgcgctgcagccattatccgaaccatccgctgtg  gtataccctgtgcgatcgctatggcctgtatgtggtggatgaagcgaacattgaaacccatggcatggtgccgatgaaccgcctga  ccgatgatccgcgctggctgccggcgatgagcgaacgcgtgacagcgcgatcgcaaccatccgagcgtgattatttggagcctgg  gcaacgaaagcggccatggcgcgaaccatgatgcgctgtatcgctggattaaaagcgatccgagccgcccggtgcagtatgaagg  cggcggcgcggataccaccgcgaccgatattatttgcccgatgtatgcgcgcgtggatgaagatcagccgtttccggcggtgccgaa  atggagcattaaaaaatggctgagcctgccgggcgaaacccgcccgctgattctgtgcgaatatgcgcatgcgttgggcaacagcctg  ggcggctttgcgaaatattggcaggcgtttcgccagtatccgcgcctgcagggcggctttgtgtgggattgggtggatcagagcctgatta  aatatgatgaaaacggcaacccg | 1576 |
| MKUV-Tra52 | MN172244 | aacggcgaatggcgctttgcgtggtttccggcgccggaagcggtgccggaaagctggctggaatgcgatctgccgga  agcgaccgtggtggtgccgagcaactggcagatgcatggctatgatgcgccgatttataccaacgtgacctatccgatta  ccgtgaacccgccgtttgtgccgaccgaaaacccgaccggctgctatagcctgacctttaacgtggatgaaagctggctgc  aggaaggccagacccgcattatttttgatggcgtgaacagcgcgtttcatctgtggtgcaacggccgctgggtgggctatggc  caggatagccgcctgccgagcgaatttgatctgagcgcgtttctgcgcgcgggcgaaaaccgcctggcggtgatggtgctg  cgctggagcgatggcagctatctggaagatcaggatatgtggcgcatgagcggcatttttcgcgatgtgagcctgctgcataa  accgaccacccagattagcgattttcatgtggcgacccgctttaacgatgattttagccgcgcggtgctggaagcggaagtgca  gatgtgcggcgaactgcgcgattatctgcgcgtgaccgtgagcctgtggcagggcgaaacccaggtggcgagcggcaccgc  gccgtttggcggcgaaattattgatgaacgcggcggctatgcggatcgcgtgaccctgcgcctgaacgtggaaaacccgaaac  tgtggagcgcggaaattccgaacctgtatcgcgcggtggtggaactgcataccgcggatggcaccctgattgaagcggaagcgt  gcgatgtgggctttcgcgaagtgcgcattgaaaacggcctgctgctgctgaacggcaaaccgctgctgattcgcggcgtgaaccgc  catgaacatcatccgctgcatggggtgatggatgaacagaccatggtgcaggatattctgctgatgaaacagaacaactttaacgcg  gtgcgctgcagccattatccgaaccatccgctgtggtataccctgtgcgatcgctatggcctgtatgtggtggatgaagcgaacattga  aacccatggcatggtgccgatgaaccgcctgaccgatgatccgcgctggctgccggcgatgagcgaacgcgtgacgttggtcagcg  cgatcgcaaccatccgagcgtgattatttggagcctgggcaacgaaagcggccatggcgcgaaccatgatgcgctgtatcgctggatta  aaagcgatccgagccgcccggtgcagtatgaaggcggcggcgcggataccaccgcgattaatattatttgcccgatgtatgcgcgcgtg  gatgaagatcagccgtttccggcggtgccgaaatggagcattaaaaaatggctgagcctgccgggcgaaacccgcccgctgattctgtg  cgaatacatgcatgcgttgggcaacagcctgggcggctttgcgaaatattggcaggcgtttcgccagcctccgcgcctgcagggcgccttt  gtgtgggattgggtggatcagagcctgattaaatatgatgaaaacggcaacccg | 1577 |
